# Autoantibodies against Neurologic Antigens in Nonneurologic Autoimmunity

**DOI:** 10.4049/jimmunol.1801295

**Published:** 2019-03-01

**Authors:** Panos Stathopoulos, Anne Chastre, Patrick Waters, Sarosh Irani, Miriam L. Fichtner, Erik S. Benotti, Joel M. Guthridge, Jennifer Seifert, Richard J. Nowak, Jane H. Buckner, V. Michael Holers, Judith A. James, David A. Hafler, Kevin C. O’Connor

**Affiliations:** * Department of Neurology, Yale School of Medicine, New Haven, CT 06511;; † Department of Immunobiology, Yale School of Medicine, New Haven, CT 06511;; ‡ Oxford Autoimmune Neurology Group, Nuffield Department of Clinical Neurosciences, University of Oxford, Oxford OX1 2JD, United Kingdom;; § Arthritis and Clinical Immunology, Oklahoma Medical Research Foundation, Oklahoma City, OK 73104;; ¶ Oklahoma Clinical and Translational Science Institute, University of Oklahoma Health Sciences Center, Oklahoma City, OK 73104;; ‖ Department of Medicine, University of Colorado School of Medicine, Aurora, CO 80045; and; # Translational Research Program, Benaroya Research Institute at Virginia Mason, Seattle, WA 98101

## Abstract

The aim of this study was to test whether autoantibodies against neurologic surface Ags are found in nonneurologic autoimmune diseases, indicating a broader loss of tolerance. Patient and matched healthy donor (HD) sera were derived from four large cohorts: 1) rheumatoid arthritis (RA) (*n* = 194, HD *n* = 64), 2) type 1 diabetes (T1D) (*n* = 200, HD *n* = 200), 3) systemic lupus erythematosus (SLE) (*n* = 200, HD *n* = 67; neuro-SLE *n* = 49, HD *n* = 33), and 4) a control cohort of neurologic autoimmunity (relapsing-remitting multiple sclerosis [MS] *n* = 110, HD *n* = 110; primary progressive MS *n* = 9; secondary progressive MS *n* = 10; neuromyelitis optica spectrum disorders *n* = 15; and other neurologic disorders *n* = 26). Screening of 1287 unique serum samples against four neurologic surface Ags (myelin oligodendrocyte glycoprotein, aquaporin 4, acetylcholine receptor, and muscle-specific kinase) was performed with live cell–based immunofluorescence assays using flow cytometry. Positive samples identified in the screening were further validated using autoantibody titer quantification by serial dilutions or radioimmunoassay. Autoantibodies against neurologic surface Ags were not observed in RA and T1D patients, whereas SLE patients harbored such autoantibodies in rare cases (2/200, 1%). Within the CNS autoimmunity control cohort, autoantibodies against aquaporin 4 and high-titer Abs against myelin oligodendrocyte glycoprotein were, as expected, specific for neuromyelitis optica spectrum disorders. We conclude that neurologic autoantibodies do not cross disease barriers in RA and T1D. The finding of mildly increased neurologic autoantibodies in SLE may be consistent with a broader loss of B cell tolerance in this form of systemic autoimmunity.

## Introduction

The presence of serum autoantibodies is commonly associated with autoimmune diseases including rheumatoid arthritis (RA), type 1 diabetes (T1D), and systemic lupus erythematosus (SLE) (1–3). These autoantibodies include antinuclear Abs (ANA), Abs to dsDNA, Sm, nuclear ribonucleoprotein, Ro, La, and phospholipids in SLE (1); rheumatoid factor and Abs to cyclic citrullinated peptides in RA (2); and Abs to insulin, glutamic acid decarboxylase (GAD65), the 40K fragment of tyrosine phosphatase (IA2), and zinc transporter 8 (ZnT8) in T1D (3). Autoantibodies against cell surface Ags associated with the nervous system have been linked to several neurologic diseases (4). In particular, autoantibodies to aquaporin 4 (AQP4) are associated with neuromyelitis optica spectrum disorders (NMOSD) (5, 6). Autoantibodies to myelin oligodendrocyte glycoprotein (MOG) are associated with a relatively stereotyped set of clinical presentations including recurrent optic neuritis, acute disseminated encephalomyelitis, pediatric acquired demyelinating syndrome, and NMOSD without AQP4 autoantibodies (7–13). Finally, autoantibodies to the acetylcholine receptor (AChR) and to muscle-specific kinase (MuSK) are associated with myasthenia gravis (MG) (14, 15).

Although most autoantibodies exhibit varying degrees of specificity toward one autoimmune disease, the presence of the same autoantibodies in different autoimmune diseases could reflect common genetic influences, common epidemiologic relationships, or other common mechanisms contributing to dysregulated B cells that produce autoantibodies (16). One such example is the presence of nonneurologic autoantibodies such as ANA and dsDNA in both nonneurologic (SLE) and neurologic (NMOSD) autoimmunity, possibly reflecting common elements of pathophysiology between SLE and NMOSD (17). In the current study, we sought to expand cross-disease autoantibody exploration to include four validated neurologic autoantibodies (MOG, AQP4, AChR, and MuSK) in nonneurologic autoimmune diseases. In particular, we examined both organ-specific and systemic nonneurologic autoimmune diseases (T1D, RA, and SLE), while simultaneously controlling for the neuro-SLE subtype. We additionally examined a control cohort of neurologic autoimmune diseases (multiple sclerosis [MS], NMOSD, and other neurologic disorders [OND]). Detection of neurologic autoantibodies in nonneurologic autoimmune diseases would indicate that a loss of tolerance toward nervous system surface Ags is a global event across neurologic and nonneurologic autoimmunity. It would further indicate that neurologic and nonneurologic autoimmune diseases share pathogenic elements that lead to similarly dysregulated, autoantibody-producing B cells. This study leverages four large and well-characterized patient cohorts with matched control samples from major United States academic centers that specialize in each respective disease. The strength of this multicenter study is the combination of well-curated specimens with thorough, state-of-the-art autoantibody detection techniques. Data from this study are anticipated to establish baseline rates of neurologic autoantibodies across several diseases.

## Materials and Methods

### Patients and healthy donors

This study was performed in accordance with the Helsinki Declaration under protocols that were approved by the institutional review boards of the Benaroya Research Institute, the University of Colorado, the Oklahoma Medical Research Foundation (OMRF), and the Yale University School of Medicine. Written and informed consent was obtained from all subjects or legal representatives before any study-specific procedures. Specimens received at Yale from other institutions were deidentified. Four cohorts were examined (RA, T1D, SLE and neurologic [MS, NMOSD, OND]). As the cohorts originated from different sites, site-specific, matched healthy controls were included for each cohort. Comprehensive clinical and demographic information were available for all individuals.

The RA samples were derived from the Studies of the Etiology of RA cohort, established at the University of Colorado (18). RA patients met at least four 1987 American College of Rheumatology (ACR) RA classification criteria (19). The T1D cohort was established at Benaroya Research Institute. T1D patients met the 2010 American Diabetes Organization criteria (20). The SLE cohort was derived from the Lupus Family Registry and Repository, established at OMRF (21, 22). SLE patients met at least four American College of ACR SLE classification criteria (23). The SLE-neuro subset included patients who met the ACR classification criteria for neuropsychiatric lupus syndromes (24). The neurologic (MS, NMOSD, OND) cohort was established at the Yale University School of Medicine, Department of Neurology. MS patients met the 2010 McDonald criteria, and NMOSD patients met the 2015 NMOSD consensus criteria (6, 25). OND and MG patient samples were collected from the Yale Multiple Sclerosis and Neuromuscular Clinics. MG diagnosis was based on both clinical criteria and autoantibody status.

### Plasmid constructs and transient transfection of human embryonic kidney cells

Expression vectors were constructed by cloning cDNA encoding full-length human MOG (26), AQP4-M1 isoform (27, 28), or rapsyn (29) into pEGFP-N plasmid vectors (Clontech, Mountain View, CA); these vectors were kindly provided by Drs. M. Reindl of the University of Innsbruck, S. Hinson and V. Lennon of the Mayo Clinic, and D. Beeson of the University of Oxford, respectively. The cDNA encoding human AChR α-, β-, δ-, or ε-subunits (29) was cloned into pcDNA3.1-hygro plasmid vectors (Invitrogen, CA) and cDNA encoding human full-length MuSK (30) was cloned into pIRES2-EGFP plasmid vector (Clontech). AChR and MuSK vectors were kindly provided by Drs. D. Beeson and A. Vincent of the University of Oxford, respectively.

Human embryonic kidney 293T cells (HEK293T) (CRL-11268; American Type Culture Collection, Manassas, VA) were transfected as previously described (29, 31). Briefly, HEK293T were transiently transfected using polyethylenimine (PEI) for 16 h with plasmid vectors encoding either MOG-EGFP, AQP4-EGFP, adult AChR, and rapsyn-EEGFP (with α-, β-, δ-, ε- subunits and rapsyn-EGFP in a ratio of 2:1:1:1:1), or MuSK-EGFP. Transfections using PEI with an EGFP-only vector and mock transfections using PEI with PBS comprised negative controls. After a wash step with PBS and a 24-h incubation period, live cells were used for cell-based immunofluorescence assay Ab measurements.

### Robot-assisted cell-based immunofluorescence assay screening

Serum was procured at enrollment and stored at −20°C/−80°C. Non-Yale serum samples were shipped on dry ice. Assays were performed on freshly thawed serum samples in blinded batches. Sera from all participants were screened for the presence of autoantibodies with a robot-assisted cell-based immunofluorescence assay as previously described (29, 31, 32). Briefly, transiently transfected HEK293T were trypsinized, washed, and resuspended at 10^6^ cells/ml in PBS containing 1% FCS and 1 mM EDTA (termed FACS buffer). Cells were rotated for 1 h at 4°C, washed and resuspended at 10 × 10^6^ cells/ml in PBS, then stained with LIVE/DEAD stain (L34972; Thermo Fisher Scientific, Waltham, MA) and incubated for 30 min at room temperature. Cells were washed after staining and resuspended at 200,000 cells/60 μl in FACS buffer. All further steps were performed in 96-well plates using the Biomek FXP Laboratory Automation Workstation, a platform that uses robotic automation (Beckman Coulter, Brea, CA) to optimize reproducibility across experiments. For each experiment, 160 serum samples were simultaneously tested in a blinded manner on the following six conditions: MOG-, AQP4-, AChR-, MuSK-, EGFP-, and nontransfected cells (PBS). Each patient serum (at a 1:50 dilution in FACS buffer) was mixed with 200,000 cells and incubated for 1 h at 4°C. Cells were subsequently washed and incubated with secondary Ab (at a 1:1000 dilution in FACS buffer) Alexa Fluor 647 rabbit anti-human IgG Fcγ fragment specific, which specifically does not recognize IgM, (309-605-008; Jackson ImmunoResearch Laboratories, West Grove, PA) for 1 h at 4°C. Finally, cells were washed, resuspended in 200 μl of 2 mM EDTA PBS, and analyzed on a BD LSR II cytometer using the high-throughput screening plate reader (BD Biosciences, San Jose, CA). After exclusion of doublets, live cells were gated on the PE–Texas Red channel. The fraction of transfected cells was measured in the FITC channel, and IgG bound was measured in the Alexa Fluor 647 channel. For each experiment, the Alexa Fluor 647 gate was set on a sample of FACS buffer incubated with Ag-transfected cells.

**FIGURE 1.**
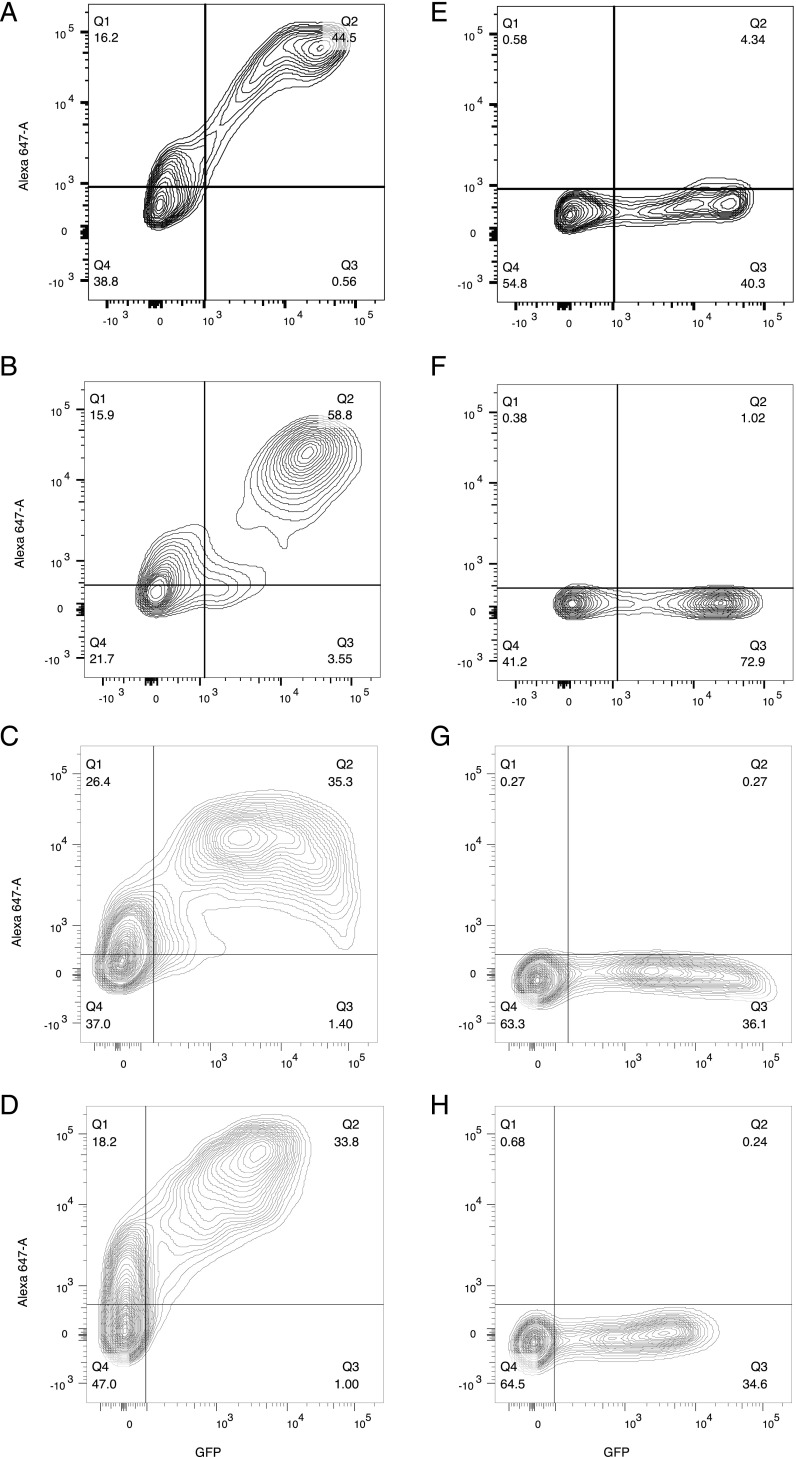
Representative cell-based assay flow cytometry plots. The *x*-axis represents GFP fluorescence intensity and consequently the fraction of HEK cells transfected with Ag (MOG, AQP4, AChR, or MuSK). The *y*-axis represents Alexa Fluor 647 fluorescence intensity, which corresponds to secondary anti–human IgG Fc Ab binding and consequently primary Ab binding to the respective Ag. (**A**–**D**) Positive serum samples at a 1:50 dilution; (**E**–**H**) negative serum samples at a 1:50 dilution; (A and E) MOG-EGFP transfection; (B and F) AQP4-EGFP transfection; (C and G) AChR-EGFP transfection; (D and H) MuSK-EGFP transfection.

Results were calculated as the difference (Δ) in the percentage of transfected cells that bound secondary Ab (termed positive cells) (31) as follows: Δ% positive cells = (% frequency of positive Ag-transfected cells (quadrant [Q]2)/% frequency of Ag-transfected cells (Q2+Q3) – (% frequency of positive EGFP-transfected cells (Q2)/% frequency of EGFP-transfected cells [Q2+Q3]) (Fig. 1). Every plate included, as positive controls, sera from patients previously determined by routine diagnostics to harbor autoantibodies against MOG, AQP4, AChR, or MuSK. Values (Δ% IgG-bound/-positive cells) both >5 SD above the mean of the matched healthy donor (HD) subjects of the respective patient cohort (RA, T1D, SLE, neuro-SLE, or neurologic [MS, NMOSD, OND]), and greater than a value (Δ% IgG-bound/-positive cells) of at least 10% were considered positive in the robot-assisted cell-based immunofluorescence assay screening.

To calculate inter- and intra-assay variability coefficients and the transfection efficiency variance for the cell-based immunofluorescence assay, each Ag (MOG, AQP4, AChR, MuSK) was transfected six times into HEK293T. Four positive control sera (each positive for MOG, AQP4, AChR, and MuSK autoantibodies, respectively) were run in triplicate over cells transfected with their matching Ag. The Δ% of positive cells was calculated for each individual condition. Coefficients of variation were calculated as the SD:mean × 100. Transfection efficiency was quantified as the percentage of transfected cells by means of EGFP fluorescence.

### Validation of screening positives

For all samples determined to be positive in the screening assay and for which serum was available, validation was performed. For the screening cell-based immunofluorescence assays, we used a cellular system that relied on transfection with the Ag in question and control transfection with EGFP; this system involves application of the same serum sample to two separate wells: one with Ag-transfected wells and one with EGFP-transfected wells. This method offers the advantage of better controlling for potential changes of the cell surface with transfection and hyperexpression and is suitable for screening to maximize sensitivity. This approach, however, can produce false positives because of nonspecific binding of serum Abs to both Ag-transfected and -nontransfected cells (respectively Q2 and Q1, Fig. 1, for Ag transfection) but not to EGFP-transfected and -nontransfected cells (again, Q2 and Q1, but for EGFP-only transfection). Such a phenomenon can produce a Δ% positive cells value above the cutoff, reflecting a binding artifact with Ag-transfected cells but not with EGFP-transfected cells. Calculating the ratio of Δ% positive cells (described above) does not control for such an artifact. We therefore performed our validation using serial dilution cell-based immunofluorescence assays measuring Δ mean fluorescence intensity (MFI) for both MOG and AQP4. For AChR and MuSK we used an RIA. We reasoned that a two-level experimental procedure for the testing of neurologic autoantibodies is most accurate, with the first level offering thorough control for transfection-induced hyperexpression and the second level eliminating false positives.

For MOG, validation was performed by serial 3-fold serum dilution cell-based immunofluorescence assays. Sera that were positive in the screening cell-based immunofluorescence assays and additional HD sera (because of limited cohort serum quantity, additional HD sera were acquired from a biorepository at Yale) were manually assayed (no robotic assistance) on the flow cytometry MOG cell-based immunofluorescence assay (31). Positivity was determined by a ΔMFI index; live, single HEK293T were first gated into transfected and nontransfected based on their EGFP expression, and subsequently the mean Alexa Fluor 647 fluorescence intensity for transfected and nontransfected cells was calculated. ΔMFI was defined as Alexa Fluor 647 MFI in MOG-transfected cells minus Alexa Fluor 647 MFI in nontransfected cells. The ΔMFI positivity cutoff for each dilution was set at the HD mean plus 5 SD; ΔMFI values above the cutoff were considered positive. The titer was defined as the highest dilution yielding positive ΔMFI.

For AQP4, validation was also performed by serial 3-fold serum dilution cell-based immunofluorescence assays. Sera that were positive in the screening cell-based immunofluorescence assays and additional HD sera (because of limited cohort serum quantity, additional HD sera were acquired from the Yale Neurology Biorepository) were manually assayed on the flow cytometry AQP4 cell-based immunofluorescence assay (31). Positivity was determined by a ΔMFI index; live, single HEK cells were first gated into transfected and nontransfected based on their EGFP expression, and subsequently the mean Alexa Fluor 647 fluorescence intensity for transfected and nontransfected cells was calculated. ΔMFI was defined as Alexa Fluor 647 MFI in AQP4-transfected cells, minus Alexa Fluor 647 MFI in nontransfected cells. The ΔMFI positivity cutoff for each dilution was set at the HD mean plus 5 SD; ΔMFI values above the cutoff were considered positive. The titer was defined as the highest dilution yielding positive ΔMFI.

For AChR and MuSK, validation was performed by RIA using 5 μl of serum, which is the established standard of serological MG diagnosis in clinical practice (14, 15). Results are reported as the Δ cpm between sample and background. The cutoff value was calculated for every experiment from the HD mean plus 3 SD (33). Because of limited cohort serum quantity, additional HD sera were acquired from the Oxford Autoimmune Neurology Diagnostic Laboratory Biorepository. Samples above the cutoff values were tested in additional confirmatory experiments using 1 μl of serum instead of 5 μl, and with 5 μl of serum and half the amount of Ag; a sample was regarded as a validated positive if it remained above the cutoff in both repetitions.

### Data analysis

Data were plotted, presented, and analyzed with Prism version 6.0 software (GraphPad Prism 6.0, San Diego, CA).

## Results

### Patients and HDs

A total of 1287 patients and HDs from four United States–based medical centers were enrolled. The number of study participants in each cohort (RA, T1D, SLE, and neurologic [MS, NMOSD, OND]) are summarized in Table I, along with the number of immunotherapy-naive (at the time of sample acquisition) patients in each cohort. All four cohorts included site-specific, matched, healthy controls. The MS and NMOSD cohort included OND patients as disease controls; their diagnoses are listed in Supplemental Table I. In addition, an MG cohort (both AChR autoantibody positive, *n* = 20, and MuSK autoantibody positive, *n* = 20) from the Yale Myasthenia Gravis Clinic was included for assay controls.

**Table I.** Patient cohorts

Cohort	Patients (N)	Immunotherapy Naive	HD (N)
RA (University of Colorado)	194	4	64
T1D (Benaroya Research Institute)	200	200*^a^*	200
SLE (OMRF)	200	20*^b^*	67
SLE-Neuro (OMRF)	49	6	33
RRMS (Yale School of Medicine)	110	83	110
PPMS (Yale School of Medicine)	9	8	
SPMS (Yale School of Medicine)	10	8	
NMOSD (Yale School of Medicine)	15	2	
OND (Yale School of Medicine)	26	9	
Total	813	340	474

^a^ In 66/200 (33%) of these patients a recent diagnosis (within 1 y of sample acquisition) was made.

^b^ Including the AQP4 autoantibody–positive but not the AChR autoantibody–positive patient.

SPMS, secondary progressive MS; PPMS, primary progressive MS.

### Robot-assisted cell-based immunofluorescence assay screening

**FIGURE 2.**
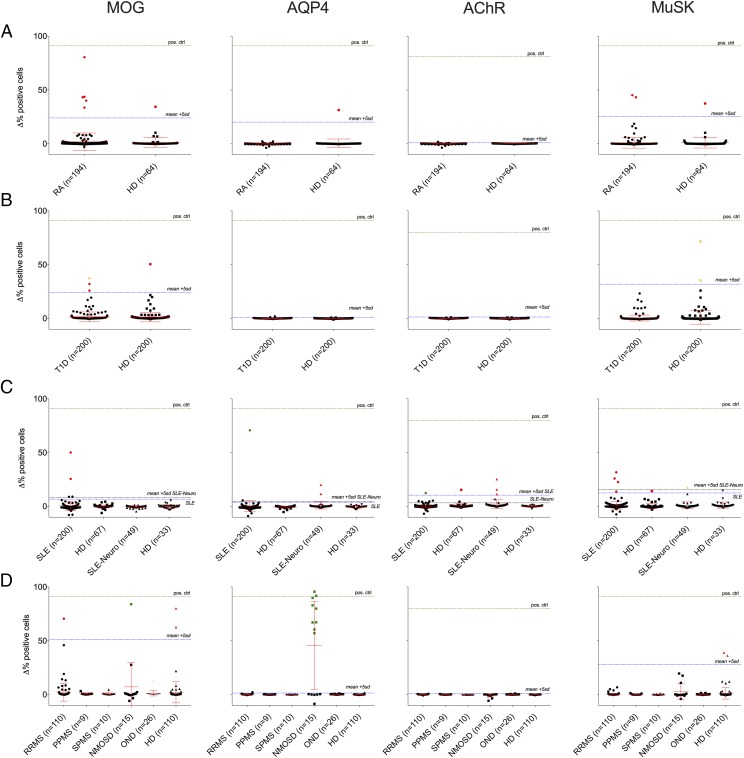
Robot-assisted cell-based immunofluorescence assay screening and validation results for the RA (**A**), T1D (**B**), SLE (**C**), and neurologic (MS, NMOSD, OND) (**D**) cohorts. The *y*-axis represents Δ in percentage of positive cells. Total Δ% positive cells values both >5 SD above the mean of the HD subjects of the cohort in question and greater than a Δ% positive cells value of at least 10% were considered positive in the robot-assisted cell-based immunofluorescence assay screening. Green dots represent positives from the screening assay that were verified in validation experiments, red dots represent positives that were not verified, and yellow dots represent samples in which serum volume was not adequate for validation experiments.

Representative cell-based immunofluorescence assay flow cytometry plots for all neurologic Ags are shown in Fig. 1. Robot-assisted screening for neurologic autoantibodies was performed on all 1287 serum specimens that encompassed the four cohorts using a cell-based immunofluorescence assay (Fig. 2). Inter- and intra-assay variability coefficients as well as transfection rates for the cell-based immunofluorescence assay are reported in Supplemental Table II. Cell-based immunofluorescence assay cutoffs of HD mean + 5 SD for the different Ags and the different cohorts were as follows: (RA: 24.0 [MOG], 20.2 [AQP4], 1.0 [AChR], 25.5 [MuSK]; T1D: 23.5 [MOG], 1.1 [AQP4], 1.4 [AChR], 32.4 [MuSK]; SLE: 6.7 [MOG], 3.9 [AQP4], 10.5 [AChR], 12.6 [MuSK]; SLE-neuro: 8.5 [MOG], 4.7 [AQP4], 3.4 [AChR], 15.8 [MuSK]; neurologic [MS, NMOSD, OND]: 51.2 [MOG], 1.5 [AQP4], 1.0 [AChR], 28.0 [MuSK]). In the RA cohort, five patients and one HD were positive for MOG autoantibodies, one HD was positive for AQP4 autoantibodies, and two patients and one HD were positive for MuSK autoantibodies. In the T1D cohort, three patients and one HD were positive for MOG autoantibodies, and two HDs were positive for MuSK autoantibodies. In the SLE cohort, two SLE patients were positive for MOG autoantibodies; one SLE patient and two SLE-neuro patients were positive for AQP4 autoantibodies; one SLE patient, one HD, and three SLE-neuro patients were positive for AChR autoantibodies; and four SLE patients, one HD, and one SLE-Neuro patient were positive for MuSK autoantibodies. Finally, in the neurologic [MS, NMOSD, OND] cohort, one relapsing-remitting MS (RRMS) patient, one NMOSD patient, and two HDs were positive for MOG autoantibodies; nine NMOSD patients were positive for AQP4 autoantibodies; and two HDs were positive for MuSK autoantibodies. Of note, none of the OND patient sera, including 10 Susac syndrome, two autoimmune encephalitis (including one *N*-methyl-d-aspartate glutamate receptor autoantibody-positive encephalitis), two stiff person syndrome, and two acute disseminated encephalomyelitis patients (Supplemental Table I) were positive for any of the four neurologic autoantibodies tested.

**FIGURE 3.**
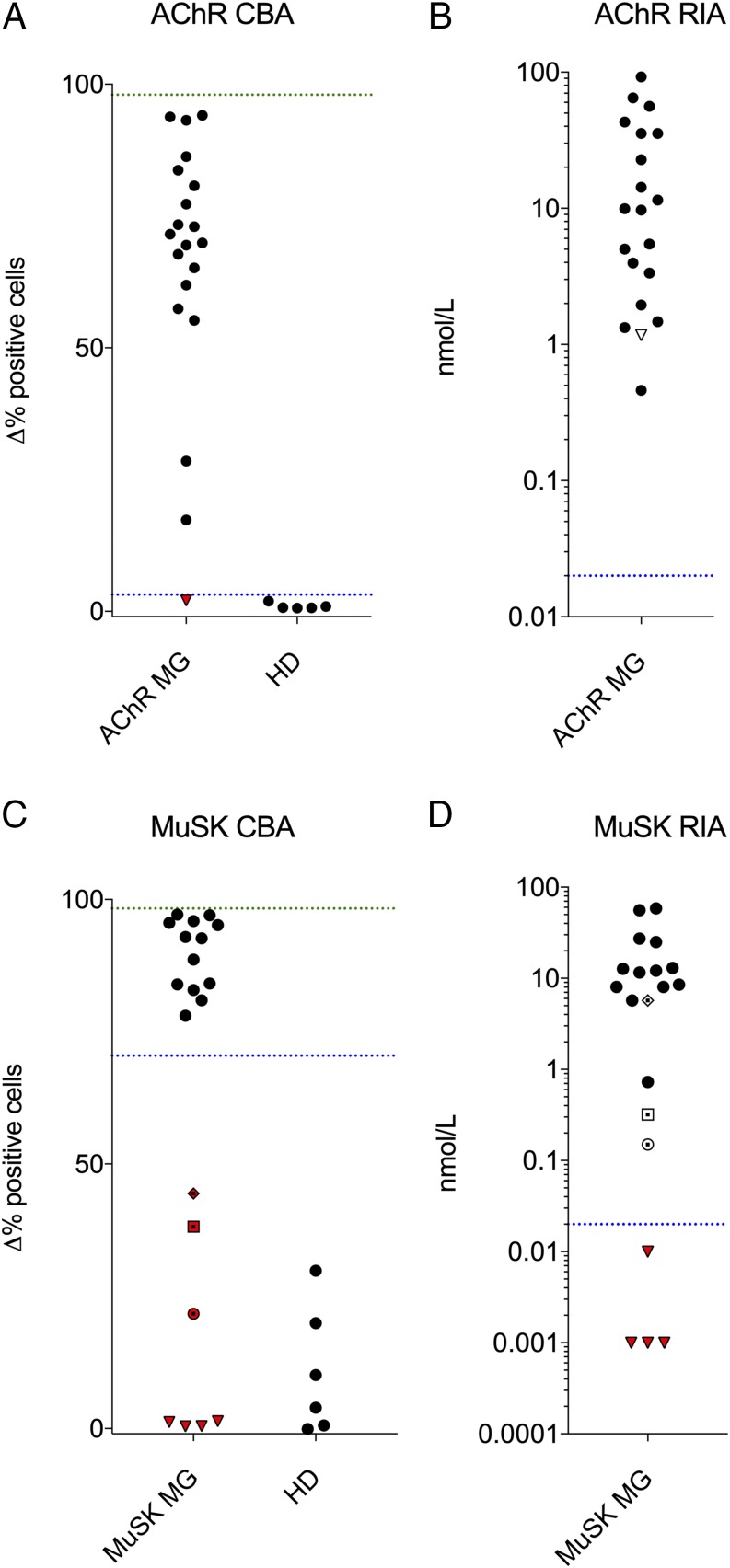
Cell-based assay (**A** and **C**) and RIA (**B** and **D**) data showing AChR and MuSK serum autoantibody-positive and -negative controls. (B and C) The *y*-axis represents the Δ in percentage of positive cells. Values (Δ% positive cells) both >5 SD above the mean of the HD subjects (AChR 3.17; MuSK 70.5; blue dotted line) and greater than a value (Δ% positive cells) of at least 10% were considered positive. The green dotted line represents positive control mAbs 637 (AChR) and 4A3 (MuSK). Each symbol represents the mean of a duplicate experiment. (B and D) The *y*-axis represents nmol/l. The clinically determined cutoff for the commercial RIA was 0.02 nmol/l for both AChR and MuSK. Triangles, squares, diamonds, and empty circles represent the samples depicted with the same symbol in the cell-based immunofluorescence assay graphs (A and C). Red symbols in all graphs represent negative patient samples.

In the initial screening, performance controls for the AQP4 and MOG cell-based immunofluorescence assays were present in the NMOSD cohort. However, no such controls were present for AChR and MuSK. Therefore, to confirm the performance of the AChR and MuSK cell-based immunofluorescence assays, we used a set of clinical samples known to be both positive and negative for MG autoantibodies. In particular, AChR and MuSK cell-based immunofluorescence assays were performed in sera derived from a Yale MG cohort using mAbs 637 (AChR) and 4A3 (MuSK) as positive controls (31). Results showed that 19 out of 20 AChR and 13 out of 20 MuSK MG patients were positive for AChR and MuSK autoantibodies, respectively (Fig. 3). These results were consistent with those acquired using a commercial RIA; one AChR patient that was negative in the cell-based immunofluorescence assay was low positive in the RIA. In addition, four of the seven MuSK MG patients that were negative in the cell-based immunofluorescence assay were also negative in the RIA. Of note, these four patients were shown to harbor MuSK autoantibodies by commercial RIA at diagnosis at a timepoint prior to acquisition of the samples tested in the cell-based immunofluorescence assay.

### Validation of positive screening results

**FIGURE 4.**
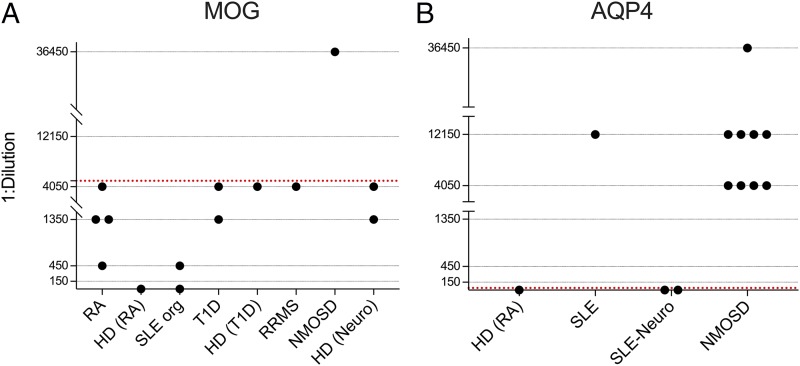
(**A**) Positive MOG autoantibody screening result validation by serial dilution cell-based immunofluorescence assay. The *y*-axis represents the maximal dilution of the sample that was positive for MOG autoantibodies, and the *x*-axis represents separate samples that were initially positive during screening in each group (HD groups of different cohorts are presented separately). Data points with a value of *y* = 0 were negative at a 1:150 dilution. The red dotted line represents the dilution cutoff for positivity. (**B**) Positive AQP4 autoantibody screening result validation by serial dilution cell-based immunofluorescence assay. The *y*-axis represents the maximal dilution of the sample that was positive for AQP4 autoantibodies, and the *x*-axis represents separate samples that were initially positive during screening per group. Data points with a value of *y* = 0 were negative at a 1:50 dilution. The red dotted line represents the dilution cutoff for positivity.

Serum samples that were identified as autoantibody-positive in the screening assays were next tested with complementary approaches for validation purposes. The MOG and AQP4 serum autoantibodies were reexamined in titer-determining cell-based immunofluorescence assays over a wide range of dilutions (Fig. 4). The cutoff titer/dilution was set at the maximum HD-positive dilution. For MOG, as HDs were positive at dilutions up to 1:4050, the titer cutoff was set above 1:4050; samples that displayed MOG binding at a higher dilution were considered to be validated as positive. For AQP4, as no HD was positive at a dilution of 1:50, the titer cutoff was set at 1:50; samples that displayed AQP4 binding at a 1:50 dilution were considered to be validated as positive. The AChR and MuSK serum autoantibodies were reexamined using RIAs (Tables II, III). The validation assays (Supplemental Table III) showed that one NMOSD patient sample was positive for MOG autoantibodies (at a 1:36,450 dilution). Autoantibodies against AQP4 were present in 60% (9/15) of NMOSD patients and one SLE patient. A second SLE patient was found to harbor AChR autoantibodies. None of the serum samples that were initially positive for MuSK in the screening assays were validated using the RIA. In four instances, there was not enough serum remaining for validation of positive screening results: one T1D patient sample initially positive for MOG autoantibodies, two HD samples initially positive for MuSK autoantibodies, and one SLE-neuro patient sample initially positive for MuSK autoantibodies.

**Table II.** AChR-positive screening result validation by RIA

Identification/Specimen	Δ% Positive Screening CBA	Δ cpm RIA
SLE	12.5	**2,469**
HD (SLE)	15.5	−323
SLE-neuro 1	25.3	−345
SLE-neuro 2	15.7	−20
SLE-neuro 3	11.4	−246
Positive control CBA	80.0	NA
Negative control CBA	0.3	NA
Positive control RIA	NA	**15,032**
Negative control RIA	NA	86

Results of validation RIAs (5 μl of serum, whole Ag) are reported. Δ cpm cutoff = 363 (mean + 3 SD).

CBA, cell-based immunofluorescence assay.

**Table III.** MuSK-positive screening result validation by RIA

Identification/Specimen	Δ% Positive Screening CBA	Δ cpm RIA
HD (T1D) 1	71.6	ND^*a*^
HD (T1D) 2	35.6	ND^*a*^
SLE 1	31.9	71
SLE 2	26.0	−68
SLE 3	22.8	−145
SLE 4	13.8	−32
HD (SLE)	14.4	−51
SLE-neuro	17.8	ND^*a*^
RA 1	45.2	−62
RA 2	43.2	−59
HD (RA)	37.5	−20
HD (MS) 1	39.0	32
HD (MS) 2	36.5	72
Positive control CBA	91.0	NA
Negative control CBA	0.2	NA
Positive control RIA	NA	**23,204**
Negative control RIA	NA	85

Results of validation RIAs (5 μl of serum, whole Ag) are reported. Δ cpm cutoff ranged from 203 to 359 (mean + 3 SD).

^*a*^Δ cpm RIA was not determined due to inadequate serum quantity.

CBA, cell-based immunofluorescence assay; NA, not applicable.

### Clinical features of patients with neurologic autoantibodies

Two SLE patients harbored autoantibodies against surface Ags of the nervous system. The first SLE patient, a 51-year-old female patient, was positive for AQP4 autoantibodies without having any signs or symptoms of NMOSD. This patient was also diagnosed with RA. The second SLE patient, a 32-year-old female patient, was positive for AChR autoantibodies, consistent with her history of concurrent MG.

In the control neurologic (MS, NMOSD, OND) cohort, 9 of 15 NMOSD patients harbored AQP4 autoantibodies, and one AQP4 autoantibody–negative patient harbored high-titer (1:36,450) MOG autoantibodies. All NMOSD patients met the 2015 NMOSD consensus criteria (6). Magnetic resonance imaging from the MOG autoantibody–positive patient’s first clinical attack showed long extensive myelitis and a pontine lesion (Supplemental Fig. 1). Relapses in this patient were no longer observed after B cell depletion with rituximab was initiated.

## Discussion

We performed robot-assisted, live cell– and flow cytometry–based immunofluorescence assays against two CNS (MOG, AQP4) and two peripheral nervous system (AChR, MuSK) surface Ags in four large and clinically well-characterized cohorts of patients with autoimmune disease (RA, T1D, SLE, and neurologic [MS, NMOSD, OND]) and matched HDs. Live cell–based immunofluorescence assays constitute a more physiologic approach for screening of serum autoantibodies. These assays offer significant advantages for autoantibody detection compared with other assays, such as ELISA; complex multimeric and membrane-bound Ags are difficult to purify for ELISA-based approaches, and moreover, cell-based immunofluorescence assays present surface Ags in their native structure and with posttranslational modifications such as glycosylation and are therefore more sensitive and specific (34). We performed our robot-assisted cell-based immunofluorescence assay screening in large batches to minimize batch-to-batch variation. We used full-length human constructs so as to better simulate naturally occurring conformations: the α-1 isoform of MOG, the M1 isoform of AQP4, the adult AChR clustered with cytosolic rapsyn, and full-length MuSK. In addition, we used a secondary Ab that does not bind to non-IgG subclasses to exclusively detect IgG Abs and minimize non-IgG false-positive results often produced by IgM (8, 28, 29, 32). Finally, we employed a second level of validation assays to confirm positive results from our initial screening approach.

Screening cell-based immunofluorescence assays revealed a higher frequency of MOG and MuSK autoantibody-positive samples compared with AChR or AQP4 (a total of 12/643 [1.9%], 8/643 [1.2%], 5/643 [0.8%], and 4/643 [0.6%] positive samples in the nonneurologic autoimmunity cohorts, respectively). The majority of positive samples in the screening cell-based immunofluorescence assays exhibited a Δ% positive cells that was less than 50%, which was rather low compared with the strongly positive samples and controls, which exceed 90 or 95%. Screening results reflect an increased tendency of MOG and MuSK toward low-to-medium (lower than 50%) IgG binding. After validation experiments, however, neurologic autoantibodies were present in only two cases of SLE. Although cross-disease autoantibodies were rare in SLE patients, such autoantibodies were not found in equally large cohorts of RA and T1D patients. Although it cannot be ruled out that this difference between SLE and RA/T1D is stochastic, it is also possible it reflects a broader reduction of self-tolerance in SLE than in RA/T1D. A broader reduction of self-tolerance in SLE would be congruous with mildly increased alternate autoimmunity, defined by the presence of at least one autoantibody primarily associated with another autoimmune disease, in SLE compared with RA and T1D (35). Moreover, patients with SLE have been known to harbor Abs against various nervous system Ags with or without associated clinical comorbidity (36, 37). In contrast, examination of B cell tolerance with an assay that was used to establish human central (bone marrow) and peripheral (blood) tolerance checkpoints does indicate similar loss of tolerance across different autoimmune diseases including SLE, RA, and T1D (38–40). It should be pointed out, however, that in these studies, tolerance checkpoints were examined by testing new emigrant and mature naive B cell populations with ANA, LPS, insulin, and dsDNA reactivity. In contrast, our serum-based study is different in that it first tested for autoantibodies that are class switched (IgG) and most likely mutated, whereas Abs from naive cells are typically unmutated. Secondly, it tested for autoantibodies against human surface Ags that can be pathogenic rather than those that are limited to disease markers, such as ANAs.

We demonstrated the presence of AQP4 autoantibodies in an SLE patient without history of NMOSD. Several lines of evidence point to an occasional association of SLE with AQP4 autoantibody–positive NMOSD. In AQP4 autoantibody–positive NMOSD, autoantibodies that relate to SLE such as ANA and dsDNA are frequently encountered (17). In SLE, AQP4 autoantibodies and concurrent NMOSD manifestations are also reported (36, 37, 41, 42). Importantly, AQP4 autoantibodies have been found to be present in the peripheral blood of SLE patients or in a single healthy individual, both without clinical evidence of NMOSD or CNS involvement (37, 43, 44); in the case of the healthy individual, NMOSD became clinically manifested 10 y later. For disease to manifest, it may be that a second event induces blood-brain barrier permeability to allow serum AQP4 IgG and/or AQP4 autoantibody–expressing B cells to enter the CNS (45, 46). Long-term follow-up of the asymptomatic AQP4 autoantibody-positive patient in our cohort would reveal if and under what circumstances NMOSD will manifest. The second nonneurologic autoimmunity patient who was positive for a neurologic autoantibody was, again, an SLE patient who was positive for AChR autoantibodies. This patient suffered from coincident MG (a fact that was not revealed during the blinded screening). SLE and AChR MG coincidence has been previously reported (47, 48). Moreover, in the context of two SLE patients that were found to be positive for AQP4 or AChR autoantibodies, it is interesting to note that AQP4 autoantibody–positive NMOSD can coincide with AChR MG without the presence of SLE, pointing toward possible common pathogenetic elements between these two autoantibody-mediated diseases (49, 50).

By implementing a two-level autoantibody testing protocol in the neurologic autoimmunity control cohort, we were able to verify and refine findings in the newly redefined and rapidly evolving field of NMOSD. Nine out of fifteen NMOSD patients harbored AQP4 autoantibodies. In three of the nine AQP4 autoantibody–positive patients, routine serum and CSF AQP4 autoantibody testing had been negative at a timepoint prior to sampling for the current study. This fact underlines the need for repeated AQP4 testing in NMOSD as previously suggested (6, 51, 52). The frequency of AQP4 autoantibody–positive NMOSD patients (9/15, 60%) was slightly lower than what has been previously reported (28, 53, 54). It remains possible that the M1 isoform cell-based immunofluorescence assay that we used is less sensitive compared with the assay using the M23 AQP4 isoform (53). In addition, our relatively small sample size may not be adequate to accurately reflect reported measures of AQP4 cell-based immunofluorescence assay sensitivity in larger cohorts. Importantly, the primary purpose for our inclusion of a NMOSD cohort was to provide positive cell-based immunofluorescence assay controls rather than measuring assay sensitivity. Furthermore, it should be pointed out that one of the six AQP4 autoantibody–negative NMOSD patients was previously serum AQP4 autoantibody–positive on routine clinical testing but had undergone a plasma exchange and i.v. steroid treatment 3 mo prior to sampling for our study; in addition, this patient was still on an oral steroid taper when the research sample was collected. Steroid treatment has been reported to lower AQP4 titers (52) and could affect seroconversion to an AQP4 autoantibody–negative status. A second AQP4 autoantibody–negative NMOSD patient harbored CSF but not serum AQP4 autoantibodies on routine clinical testing, which is a rarely observed combination (55).

In the case of MOG reactivity, we found lower dilution (as high as 1:4050) positivity in SLE, T1D, RRMS, and RA patients, but the same positivity could be found in HDs; thus, this lower dilution positivity is unlikely to be directly associated to underlying disease mechanisms. We therefore set the cutoff at a dilution of 1:4050. In previous work, absence of cell-based immunofluorescence assay MOG reactivity in MS patients and HDs (HD *n* = 13) was reported to depend on the use of a full-length human MOG α-1 isoform and an IgG1-specific secondary Ab at serum dilutions of 1:20 (32). Other studies, however, based separation of MOG autoantibody–positive patients from MS patients and HDs on serial dilutions; such studies showed that controls (*n* = 105) were positive for MOG autoantibodies at dilutions up to 1:640 (56, 57). We, however, found that MOG autoantibody positivity of HDs could be found at dilutions as high as 1:4050, and therefore the significance of similar maximal dilution MOG autoantibodies in RA, T1D, and RRMS is not clear. The difference in cutoff between our study and previous studies (11, 58) could be explained by the fact that we tested almost 5- to 10-fold more HDs’ samples (*n* = 474) and therefore encountered rare cases of higher-dilution MOG autoantibodies in healthy individuals. Differences in cell-based immunofluorescence assay methodology between studies might also contribute to the observed difference in dilution cutoffs. Importantly, our results are in agreement with a recent report from the Biomarker Study Group, where a 1:1250 MOG autoantibody titer cutoff showed better specificity than a 1:160 cutoff for prediction of a non-MS course in a pediatric population (59). Moreover, high MOG autoantibody titers correlated with severity of presentation but not with risk of future relapse in another recent study of adult patients (60).

In our neurologic autoimmunity control cohort, only one AQP4 autoantibody–negative NMOSD patient was positive for MOG autoantibodies at a dilution higher than 1:4050 (reaching 1:36,450). Clinical and magnetic resonance imaging characteristics of the MOG autoantibody–positive NMOSD patient were similar to those previously reported (61, 62). Although this is a single case, it is interesting to note that this patient responded well to B cell depletion because resistance to B cell depletion in MOG autoantibody–positive NMOSD has been reported, albeit anecdotally because of rarity of the disease (63). A second AQP4 autoantibody–negative NMOSD patient displayed subthreshold, yet slightly elevated, serum reactivity against MOG in our screening cell-based immunofluorescence assay (Δ% IgG-bound/-positive cells 27.7, below the mean + 5 SD cutoff). It is possible that this patient originally harbored MOG autoantibodies that would have resulted in a positive measurement and that the value dropped posttreatment or because of remission (11, 12, 58, 64). In this patient, the research sample was acquired 7 mo post–rituximab treatment and while the patient was in clinical remission; it should be noted that B cell depletion did not eliminate clinical relapses. For the remaining two AQP4 and MOG autoantibody–negative NMOSD patients, the possibility of new and unidentified Ags in seronegative NMOSD and of serum autoantibodies that are below the level of sensitivity of existing assays should be considered.

We performed an extensive screen for four surface neurologic autoantibodies in 1287 well-characterized patients with nonneurologic autoimmunity and controls. We could not detect neurologic autoantibodies in the serum of RA and T1D patients. In SLE, we found, albeit in rare cases (2/200, 1%), patients who harbored neurologic autoantibodies. Because we tested large cohorts it is possible that our findings do not represent a stochastic phenomenon but rather reflect a reduction of nervous system Ag tolerance in SLE patients. In the first SLE patient, the presence of AChR autoantibodies coincided with clinical disease (MG), whereas in the second patient, presence of AQP4 autoantibodies did not coincide with clinical disease (NMOSD). In the case of asymptomatic AQP4 positivity in SLE, prospective studies can clarify whether autoantibody presence precedes clinical symptoms as shown in SLE and RA (1, 65) or merely reflects a nonpathogenic abundance of different autoantibodies, and whether additional events are required for clinical manifestations to develop. Moreover, investigations of human immunology clarifying mechanisms of self-tolerance loss in SLE that lead to production of neurologic autoantibodies will deepen our understanding of human autoimmunity and improve our ability to deliver targeted therapies. Overall, this study shows that autoantibodies against neurologic Ags are confined to specific neurologic diseases.

## Supplementary Material

Data Supplement
