# Supplementary material for: Autoantibodies against Neurologic Antigens in Nonneurologic Autoimmunity
Source: J Immunol. 2019 Mar 1;202(8):2210–9. doi: 10.4049/jimmunol.1801295 (PMC6452031; doi:10.4049/jimmunol.1801295)
Supplement: Data Supplement [file JI_1801295.zip › JI_1801295_Supplemental_Material_1.pdf]

Supplemental data to accompany manuscript:

Autoantibodies against neurologic antigens in non-neurologic autoimmunity

Contents

Table S1. OND diagnoses……………………...………………………………………… p. II

Table S2. Inter-assay and intra-assay variation coefficients and transfection efficacy for the screening CBAs………………………………………………………………… p. III

Table S3. Data summary: validated positive samples…………………………………… p. IV

Figure S1. Magnetic resonance imaging of high-titer MOG autoantibody positive patient at initial presentation………………………………………………………………… p. V

Table S1. Other neurological disease (OND) diagnoses

Diagnosis	N
Susac's syndrome	10
Autoimmune encephalitis	1
NMDA receptor positive encephalitis	1
Amyotrophic lateral sclerosis	1
Stiff person syndrome	2
Acute disseminated encephalomyelitis	2
Multiple cranial neuropathy	1
Limb-girdle muscular dystrophy	1
Inclusion body myositis	1
Neuro-Behcet's disease	1
Charcot-Marie-Tooth Disease	1
Subacute combined degeneration	1
Idiopathic neuropathy	1
Neurosarcoidosis	1
Non-specific white matter MRI lesions	1
Total	26

NMDA: N-methyl-D-aspartate

Table S2. Inter-assay variation coefficients for positive serum controls and transfection efficacy for the screening CBAs.

	MuSK serum	AQP serum	AChR serum	MOG serum
Mean Δ% positive cells TF1	95.8	73.1	75.9	95.1
Mean Δ% positive cells TF2	97.5	75.1	82.5	96.0
Mean Δ% positive cells TF3	96.0	63.9	79.5	95.1
Mean Δ% positive cells TF4	93.7	68.6	89.7	96.6
Mean Δ% positive cells TF5	96.7	68.2	79.2	95.8
Mean Δ% positive cells TF6	97.9	74.2	84.0	94.8
**Inter-assay CV%**	**1.5**	**6.2**	**5.8**	**0.7**
Intra-assay CV% TF1	0.3	2.4	0.1	0.6
Intra-assay CV% TF2	0.2	0.4	2.1	0.2
Intra-assay CV% TF3	0.1	22.1	1.0	0.7
Intra-assay CV% TF4	0.1	13.4	3.3	0.3
Intra-assay CV% TF5	1.1	7.7	0.2	0.9
Intra-assay CV% TF6	0.1	1.0	2.8	0.7
**Intra-assay CV%**	**0.3**	**7.9**	**1.6**	**0.6**
	MOG	AQP4	AChR	MuSK
Efficiency% TF1	57.5	47.4	42.0	49.4
Efficiency% TF2	51.3	41.6	39.9	41.6
Efficiency% TF3	46.0	37.6	34.5	39.4
Efficiency% TF4	52.6	45.8	46.2	45.8
Efficiency% TF5	49.0	40.4	36.8	39.6
Efficiency% TF6	58.4	47.4	45.1	48.2
**mean**	**52.5**	**43.4**	**40.8**	**44.0**
**SD**	**4.8**	**4.1**	**4.6**	**4.4**

Six replicates of the same antigen transfection (TF1-TF6) were incubated with a matching positive control serum in triplicate. Mean Δ% positive cells were calculated for each condition. Inter-assay CV% were calculated from the mean of these means and the corresponding SD. For each transfection and serum-antigen pair triplicate, an intra-assay CV% was calculated from the respective mean and SD. An average intra-assay CV% was calculated as the mean of the intra-assay coefficients for each transfection. TF: transfection, MOG: myelin oligodendrocyte glycoprotein, AQP4: aquaporin 4, AChR: acetylcholine receptor, MuSK: muscle-specific kinase, CV: coefficient of variation.

Table S3. Data summary: validated positive samples

	MOG n (%)	AQP4 n (%)	AChR n (%)	MuSK n (%)
T1D	-	-	-	-
HD (T1D)	-	-	-	-
RA	-	-	-	-
HD (RA)	-	-	-	-
SLE	-	1 (0.5)	1 (0.5)	-
HD (SLE)	-	-	-	-
SLE-Neuro	-	-	-	-
HD (SLE-Neuro)	-	-	-	-
RRMS	-	-	-	-
PPMS	-	-	-	-
SPMS	-	-	-	-
NMOSD	1 (6.7)	9 (60)	-	-
OND	-	-	-	-
HD (MS)	-	-	-	-

SLE: systemic lupus erythematosus, HD: healthy donor, T1D: type 1 diabetes, RA: rheumatoid arthritis, RRMS: relapsing-remitting multiple sclerosis, PPMS: primary progressive multiple sclerosis, SPMS: secondary progressive multiple sclerosis, NMOSD: neuromyelitis optica spectrum disorder, OND: other neurologic disorder, MOG: myelin oligodendrocyte glycoprotein, AQP4: aquaporin 4, AChR: acetylcholine receptor, MuSK: muscle-specific kinase

Figure S1

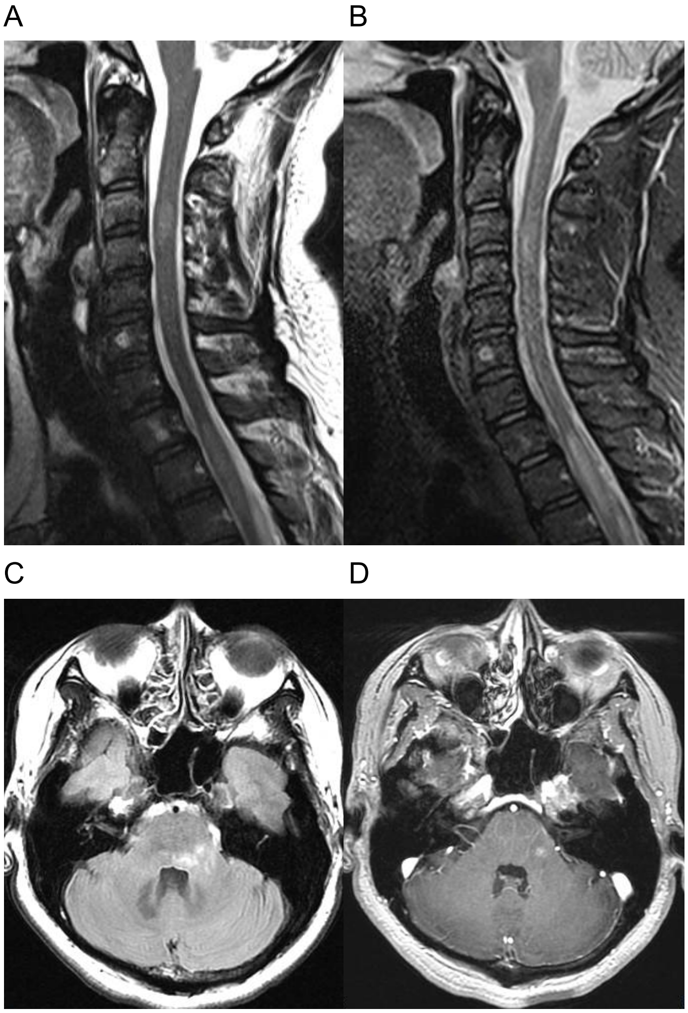

Figure S1. CNS (brain and spinal cord) magnetic resonance imaging of the high-titer MOG autoantibody positive patient at initial presentation: (A) T2-weighted images; (B) Short-TI Inversion Recovery (STIRR) images; (C) Fluid-Attenuated Inversion Recovery (FLAIR) images; (D) T1-weighted images with contrast. (A) and (B) show a long extensive myelitis and (C) and (D) show a contrast-enhancing lesion that is in contact with the brain's 4th ventricle.
